# Development and implementation of the Baltimore healthy carry-outs feasibility trial: process evaluation results

**DOI:** 10.1186/1471-2458-13-638

**Published:** 2013-07-09

**Authors:** Seung Hee Lee-Kwan, Sonja Goedkoop, Rachel Yong, Benjamin Batorsky, Vanessa Hoffman, Jayne Jeffries, Mohamed Hamouda, Joel Gittelsohn

**Affiliations:** 1 Center for Human Nutrition, Bloomberg School of Public Health, Johns Hopkins University, Baltimore, Maryland, USA; 2 Department of Health Policy & Management, Bloomberg School of Public Health, Johns Hopkins University, Baltimore, Maryland, USA

**Keywords:** Prepared food, Carry-out, Process evaluation, Food environment intervention, Low-income, African-American, Urban

## Abstract

**Background:**

Prepared food sources, including fast food restaurants and carry-outs, are common in low-income urban areas. These establishments provide foods high in calories, sugar, fat, and sodium. The aims of the study were to (1) describe the development and implementation of a carry-out intervention to provide and promote healthy food choices in prepared food sources, and (2) to assess its feasibility through a process evaluation.

**Methods:**

To promote healthy eating in this setting, a culturally appropriate intervention was developed based on formative research from direct observation, interviews and focus groups. We implemented a 7-month feasibility trial in 8 carry-outs (4 intervention and 4 comparison) in low-income neighborhoods in Baltimore, MD. The trial included three phases: 1) Improving menu boards and labeling to promote healthier items; 2) Promoting healthy sides and beverages and introducing new items; and 3) Introducing affordable healthier combo meals and improving food preparation methods. A process evaluation was conducted to assess intervention reach, dose received, and fidelity using sales receipts, carry-out visit observations, and an intervention exposure assessment.

**Results:**

On average, Baltimore Healthy Carry-outs (BHC) increased customer reach at intervention carry-outs; purchases increased by 36.8% at the end of the study compared to baseline. Additionally, menu boards and labels were seen by 100.0% and 84.2% of individuals (n = 101), respectively, at study completion compared to baseline. Customers reported purchasing specific foods due to the presence of a photo on the menu board (65.3%) or menu labeling (42.6%), suggesting moderate to high dose received. Promoted entrée availability and revised menu and poster presence all demonstrated high fidelity and feasibility.

**Conclusions:**

The results suggest that BHC is a culturally acceptable intervention. The program was also immediately adopted by the Baltimore City Food Policy Initiative as a city-wide intervention in its public markets.

## Background

Obesity is a leading cause of preventable death in the United States [1]. Being overweight or obese increases risk of diabetes, heart disease, and some cancers [2]. One factor that contributes to obesity is the food environment, with greater prevalence in settings with high availability of energy-dense prepared foods [3-5]. Further, longitudinal studies found that proximity to fast food establishments [6] and cheaper prices [7] were related to increased fast food consumption. This may also be reflective of increased expenditures on eating out in the US, which accounts for 48% of consumer food spending [8].

The high prevalence of prepared food sources and the increased consumption of prepared foods are particularly problematic for low-income urban areas. The most common food sources in these areas are small prepared food sources such as fast food restaurants and carry-outs [9-11]. Carry-outs are defined as non-franchised small local food establishments that sell ready-to-eat food and beverages for off-premise consumption [12]. They provide primarily high-calorie, high-fat and high-sodium foods [11]. These foods adversely affect nutritional status and increase risk of obesity and diet-related chronic diseases [13].

In order to address obesity concerns, the CDC recommends environmental approaches in prepared food sources [14], such as signage, price reduction, and menu labeling. These interventions have resulted in increased sales of promoted healthy foods at the prepared food source-level and improved knowledge of healthy foods and behavior changes at the customer-level [15]. While these results are promising, most prepared food source interventions have been based in middle-income, predominately Caucasian populations, and may not be generalizable to low-income, urban populations.

Another limitation with current literature is that studies focusing on prepared food sources have not conducted formative research utilizing key stakeholders when developing the interventions [16]. This is an important consideration because building rapport with storeowners can be challenging [17] and having their support often determines intervention success. In addition, formative research is needed to identify culturally appropriate foods, a key means of building consumer demand [18]. Lastly, there is a lack of research on process evaluations for prepared food source interventions.

In order to address these gaps, the Baltimore Healthy Carry-outs (BHC) intervention was developed and implemented in a low-income, urban African-American population. The goal of this paper is to describe the development and implementation of BHC and to assess its feasibility through a process evaluation. We sought to answer two main questions:

1 What methods are effective in promoting healthier new options in prepared food sources in low-income urban settings?

2 How well and to what extent was the BHC intervention implemented in terms of reach, dose and fidelity?

## Methods

### Baltimore healthy carry-outs intervention

The Baltimore Healthy Carry-outs (BHC) intervention was conducted from February to September 2011 in eight carry-outs located in low-income neighborhoods of Baltimore (Table 1). All neighborhoods were predominantly African-American (90%) and the annual median household income ranged from $20,515 - $30,597, which was much lower than the city average ($37,395) [19]. In 2010, the study areas had 144 prepared food sources. Approximately 70% of them were carry-outs, and over 50% were owned by Korean immigrants [12].

**Table 1:** Stage of development of the Baltimore healthy carry-outs trial

**Stage 1**	**Stage 2**	**Stage 3**
**Formative research**	**Development of intervention strategies**	**Implementation of pilot study**
*Apr 2009 – Dec 2010*	*Dec 2010- Apr 2011*	*Feb 2011- Sep 2011*
• In–depth interviews	• Multi-component intervention strategies	• Selection of intervention and matched comparison carry-outs
• Semi-structured questionnaires	• Materials development	• Three phase program:
• Direct observations	- Promoted foods	- Phase 1: Modified menu boards & menu labeling
• Focus groups	- Audience selection	- Phase 2: Healthy sides & beverages
• Conjoint analysis	- Target behaviors	- Phase 3: Affordable healthy combination meals
		• Process evaluation

### Carry-out sampling strategy

We stratified our selection of carry-outs by geographic location (East and West Baltimore) and race/ethnicity of storeowners (Korean-Americans and African-Americans). Four intervention carry-outs were randomly selected per strata. In addition, four comparison carry-outs were matched for the physical environment (e.g. presence of Plexiglas, lack of tables, etc.), principal types of food offered (e.g. fried chicken, sandwiches), and neighborhood characteristics (e.g. median income, % of African-American ethnicity). Comparison carry-outs located in the same neighborhood (minimum of 0.5 miles away from an intervention carry-out) were avoided since intervention strategies could have impacted the comparison group. Overall, 66% of carry-outs approached agreed to participate.

### Formative research to develop intervention strategies

Formative research was conducted to understand carry-out owners’ perceived and real barriers to serving healthy foods, community members’ rationale for current prepared food sources purchasing behaviors [20] and to determine culturally appropriate menu items and intervention materials. The research focused on (1) African-American customers (n = 50) aged ≥18 years who made purchases at prepared food sources, and (2) carry-out owners (n = 16) managing food preparation, pricing, and sales. Customers provided information on factors governing their ordering practices and which healthier options were most appealing to them. Owners discussed their business practices and perceptions of customer tastes and priorities. All study participants provided written consent and ethics approval was granted by the Johns Hopkins Bloomberg School of Public Health Institutional Review Board.

Focus groups (n = 6) were conducted to help develop and review intervention materials (e.g. poster design) and strategies (e.g. types of healthy sides). Each focus group consisted of 6–8 African American adults from the neighborhoods near the carry-outs. Participants were recruited through print material and word-of-mouth; flyers were posted at the carry-outs participating in the study. Local recreation center staff and community leaders were also involved in recruiting for the focus groups. Focus group participants each received a meal and a gift card ($15) for participating. When discussing slogans and logos to promote more nutritious foods, participants responded best to catchy and informative slogans that were not ‘wordy’. Participants also disliked the word ‘healthy’, as it invoked thoughts of ‘disgusting’, ‘not tasty’ or ‘bland’. Instead, the word ‘fresh’ was positively associated with terms including ‘homemade’, ‘delicious’, or ‘vegetables’ [20].

Concerns expressed by carry-out owners were the potential for low sales of healthier items, short shelf life and high price of fruits and vegetables, and customers’ preferences for less healthy foods [20]. In order to help overcome these perceived barriers, the study used small monetary incentives, marketing materials, and technical assistance to motivate owners to sell healthier foods. We gave owners a weekly $25 gift card for 7 months as compensation for providing sales receipts. We also provided owners with free point-of-purchase materials including menu boards and posters and expert input on healthier food preparation methods.

A forced-choice conjoint analysis questionnaire was administered to a convenience sample of customers (n = 50) recruited from two randomly selected carry-outs in the BHC study, one located in East Baltimore and the other in West Baltimore [21]. The questionnaire was used to measure the relative perceived value of specific features of a combination meal and measure the demand for the particular combination of entrée (i.e. turkey club sandwich, grilled chicken sandwich), side dish (i.e. fruit, side salad) and beverage option (i.e. diet soda, bottled water). Findings suggested that carry-out customers significantly preferred water to diet soda and favored a turkey club sandwich over a grilled chicken sandwich [21].

Two dietitians reviewed menu recipes and cooking methods at each of the study carry-outs to calculate total calories (kcal) and fat (g), using the USDA National Nutrient Database (db.nal.usda.gov). Criteria for healthy entrées and sides were modified from previously conducted healthy restaurant studies [22-24]. One serving of a healthier entrée was measured to be less than 600 kcal and 20 g of fat; one serving of a healthier side dish was determined to be less than 200 kcal and 7 g of fat. For example, a grilled chicken sandwich, containing 350 kcal with 15 g of fat, was classified as a healthier option compared to a 4 piece chicken wing meal with 780 kcal and 52 g of fat. Based on the foods offered in the carry-outs, 47 healthier items and 119 less healthy items were identified.

### Development and implementation of intervention phases

#### Phase 1: modified menu boards & menu labeling

##### Development

Carry-out menus were redesigned to highlight healthier items at intervention carry-outs. At baseline, all carry-outs offered at least two times the number of less healthy items compared to healthier items [25] and all existing promotional materials around the premises promoted fried food combo meals. Mock-up versions of intervention menus were tested in focus groups, with healthier items identified using numbered color photographs.

Focus group participants recommended using a symbol that exemplified ‘green leafy vegetables’ to indicate healthier foods, which was developed by a local designer/artist. Carry-out owners and customers agreed that this symbol was appealing, acceptable, and effectively conveyed the concept of freshness.

**Figure 1:**
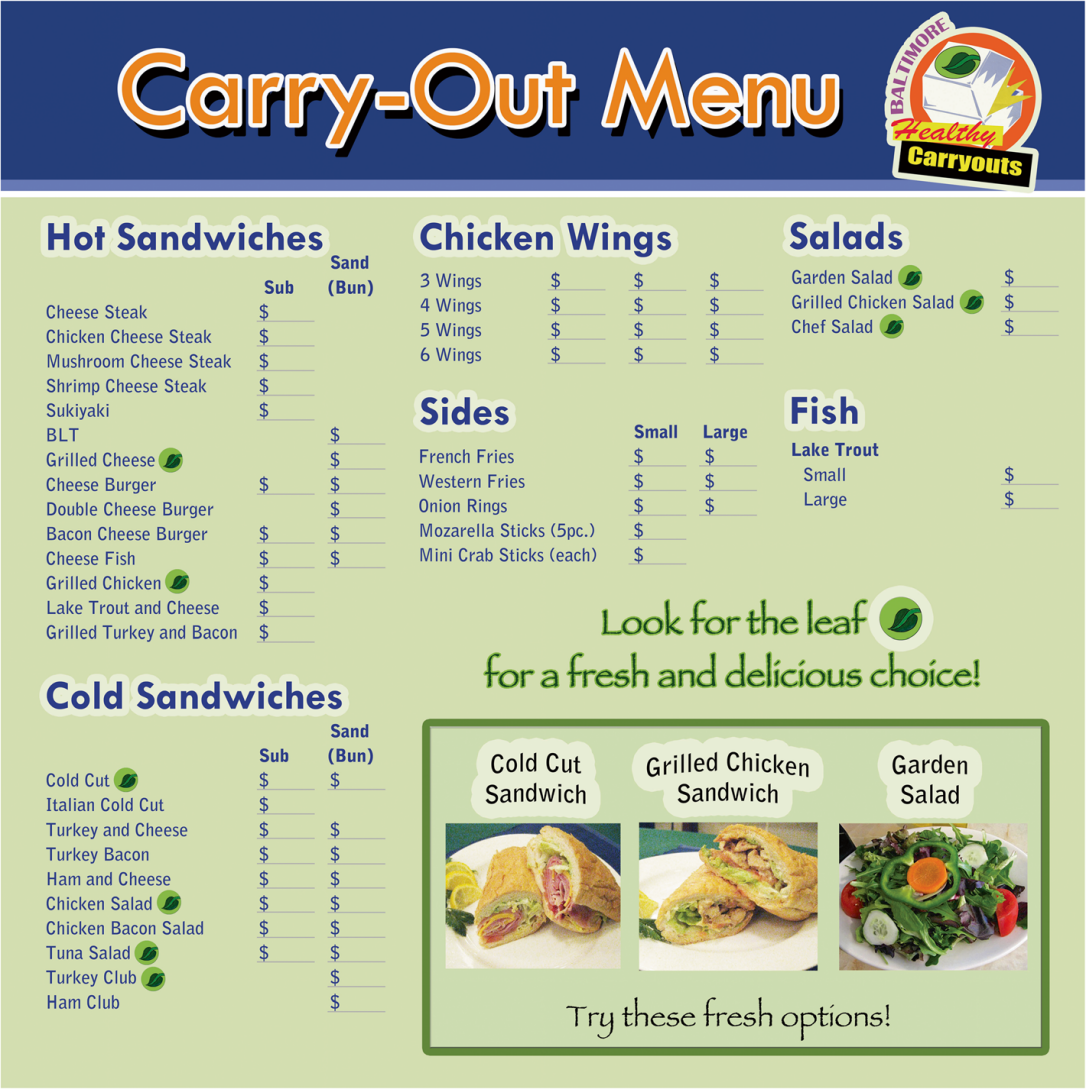
**Sample intervention carry-out menu board.** Legend: This figure is a sample menu board that was developed by Baltimore Healthy Carry-out designers to highlight healthier options at the intervention carry-outs.

The leaf symbol was added on intervention store menus adjacent to healthier items. The goal was to enhance the appeal of these items, and ultimately increase sales. Of the menu items labeled with the leaf logo, carry-out owners also selected three specific healthier options to be promoted with a photograph below the slogan, “Try these fresh options!” Menu boards were approved by store owners after several rounds of revisions (Figure 1).

##### Implementation

We replaced the existing menu boards in the intervention carry-outs with new boards and placed a variety of wall posters promoting healthier items throughout the carry-outs. Owners were consulted to determine the best location for new materials within the store, which was generally near the cash register. We also supplied owners with paper menus (~2000 per carry-out) using the same labeling strategy as the wall-mounted boards. Providing the carry-outs with paper menus was very successful and resulted in some owners independently making copies of the paper menus for continued use in their store.

Three owners were initially resistant to the idea of replacing their existing menu boards, and insisted that original boards remain next to the new ones. However, after seeing the visual appeal of the new menu boards, two of the three owners removed the old ones. Several owners stated their approval of the food photographs, which was likely an important factor for abandoning the old menus. Prices were originally typed onto the new menu boards. However, due to frequent changes in food prices we began printing laminated menus with a dry-erase option for prices.

#### Phase 2: healthy sides and beverages

##### Development

At baseline, there was a limited number of healthy sides and beverages available in carry-outs. Some carry-out owners mentioned a lack of demand for these items, while others mentioned having limited storage space. Existing healthier sides were identified (e.g. collard greens, broth-based vegetable soups, and watermelon), as well as ideas for new sides requested by community members (e.g. fruit cups, yogurt, bananas, baked potato chips, and pretzels).

##### Implementation

Existing side dishes that met our healthier criteria were promoted in addition to newly added side items. Carry-outs were given monetary incentives in the form of an initial stock supply (e.g. several boxes of yogurt and a case of individual bags of baked chips) for newly added promoted foods. At one carry-out, the owner was hesitant to add yogurt to the menu due to fear that customers would not purchase this item. As a solution, the owner agreed to temporarily hang a poster promoting yogurt to assess if any customers requested it. A few days later the owner requested that yogurt be added as a side dish to his menu offerings. After a few weeks, the owner began stocking the yogurt himself due to high demand. By providing the initial stock, we reduced the potential risk for financial loss on a new product. This strategy was used with bananas, watermelon, fruit cups, and baked chips. The stock of some items was better maintained than others, and new items that promised greater shelf-life were encouraged. Additionally, the point-of-purchasing posters were laminated to ensure longevity and quality.

All of the carry-outs offered bottled water and diet soda at baseline. We did not experience any resistance to promoting water. However, owners were doubtful that there would be an increase in sales of diet soda, despite promotion. Owners explained that they sold less than 2–3 cans/bottles per week, and only to customers with diabetes or other health conditions.

#### Phase 3: healthy affordable combination meals and changing food preparation methods

##### Development

Combination meals were offered in all study carry-outs and usually included fried chicken wings, French fries and a 32 oz soda. All combo meals included a reduced-price promotion. Our baseline findings showed that over 65% of carry-out customers purchased combo meals [25,26].

In this phase, new combo meals were tailored to each carry-out. For example, one carry-out’s only healthy entrée option was a grilled chicken sandwich, so this item was promoted in a combo meal. Another carry-out owner was concerned that customers disliked the taste of healthier foods. To address this, taste-tests were conducted with customers (n = 25) to assess the acceptance of a new entree. Subsidies were promised to carry-out owners to account for any decrease in profit they could have encountered from selling healthy combo meals for 2 months of Phase 3. For example, if a carry-out reduced the price of a healthy combo meal by $1.00 and sold 20 units, we would compensate the owners $20.

Healthier food preparation methods were also introduced in this phase. For example, one carry-out’s only cooking equipment was a deep-fryer. To allow for healthier cooking methods, we provided the owners with an indoor grill and gave a demonstration to staff on how to make grilled chicken with the equipment. Storeowner guidelines were developed regarding cost-neutral healthy alternatives to cooking (e.g. grilling) and preparation methods (e.g. reduced portion sizes and smaller amounts of high calorie condiments/sauces).

##### Implementation

We used wall posters to promote ‘fresh combo meals’ that were less than or equal in price to the original combination meal. The types of combo meals and prices were specific to each carry-out. For example, one carry-out priced a combination meal including a veggie wrap ($10) and water ($1) for $8 total. Another included a turkey sandwich ($4.50), water ($1), and baked chips ($0.50) for $5 total. All owners reduced prices voluntarily without compensation, despite our initial offer to subsidize the difference between the original and promoted price.

### Process evaluation

Process evaluation data was collected to assess whether the intervention strategies were implemented according to protocol [27,28]. Three key elements evaluated were: reach, dose received, and fidelity [27]. Reach indicated the proportion of the target population exposed to the intervention; dose received indicated the level with which customers were exposed to the intervention components; and fidelity indicated how well components of the intervention were delivered according to plan [27]. Three data collection instruments in addition to sales receipt data were used for the process evaluation (Table 2).

**Table 2:** Baltimore healthy carry-outs process evaluation instruments

**Process measures**	**Instruments**	**Administered by**	**Frequency**	**Process component**
Sales data	Receipt collection	Interventionist	Weekly	Reach
Availability of promoted foods	Carry-out visit evaluation	Process Evaluator	10 times throughout the intervention	Fidelity/Dose delivered
Placement of menu boards/posters	Carry-out visit evaluation	Process Evaluator	10 times throughout the intervention	Fidelity/ Dose delivered
Point-of-purchase materials and purchasing behaviors	Intervention exposure assessment	Interventionist	Once (at end of intervention)	Dose received

### Sales receipt collection

Every week, interventionists collected sales receipts to obtain information on reach at the carry-outs [25]. Since BHC was an environmental intervention targeting community members, it was not feasible to track reach in the traditional way (as a proportion of the total population). Following other environmental interventions, we used change in number of customers served as an indicator of reach [29]. The number of customers served was determined by counting the number of entrees sold.

### Carry-out visit evaluation form

In order to measure dose and fidelity, a modified store visit evaluation form [29] was used to assess availability of promoted entree and side dishes as well as visibility of menus and posters. Availability of promoted foods from each phase was evaluated throughout the subsequent phases. To minimize bias, a process evaluator who did not participate in the intervention conducted 10 rounds of evaluations during the intervention.

### Intervention exposure survey

To measure dose received, we conducted a modified Intervention Exposure Assessment survey [30] after the intervention conclusion at participating carry-outs. We interviewed every fifth customer (n = 101) for 10 non-consecutive days (roughly 3 days/week for 1.5 hours/day) per carry-out. Inclusion criteria were African-American individuals over 18 years of age who had been to the carry-out more than once per month over the past year. Survey questions included whether customers had seen the intervention materials and whether they had purchased promoted healthy foods because of the BHC materials.

### Statistical analysis

Descriptive statistics were performed using STATA 12.0 (STATACorp, College Station, TX) to calculate the proportion of responses for the process evaluation.

## Results

### Reach

BHC reached more customers during the intervention period than at baseline when comparing intervention carry-outs to comparison carry-outs (36.8% increase (n = 3552) at intervention carry-outs versus a 1.2% (n = 175) increase at comparison carry-outs).

### Dose received

Menu boards and menu labeling were seen by 100% and 84.2% of customers, respectively, suggesting high dose received for this component of the intervention. Posters for each of the three phases were seen 55.4%, 34.7%, and 55.4% of the time, respectively, suggesting moderate dose received (Table 3). A total of 65.3% of customers stated purchasing a particular food due to the presence of a photograph of that item on the menu board. Additionally, 42.6% of customers purchased a food due to the presence of the leaf logo, suggesting moderate dose received.

**Table 3:** Results from Baltimore healthy carry-outs process evaluation

	**Phase 1 (n = 2)**	**Phase 2 (n = 4)**	**Phase 3 (n = 4)**	**Overall (n = 10)**
		*%*		
Availability of promoted healthy entrees (frequency)	96.1	92.7	91.0	92.5
	(72/76)	(217/234)	(131/144)	(420/454)
Availability of healthy sides (frequency)	N/A	50.0	57.4	53.8
		(32/64)	(39/68)	(71/132)
Menu boards posted	100.0	100.0	100.0	100.0
Posters posted	87.5	100.0	100.0	97.5
**Customer exposure of intervention materials in intervention carry-outs (n = 101)%**				
Have you seen any of the BHC^1^ menu boards?				100.0
Have you seen any of the BHC menu labels?				84.2
Have you seen “choose the leafy logo” poster? (Phase 1)				55.4
Have you seen “eat your entrée with healthy side” poster? (Phase 2)				34.7
Have you seen any of the “choose healthy combo meal” poster? (Phase 3)				55.4
Have you purchased a food specifically because you saw the BHC fresh leaf logo?				42.6
Have you purchased a food specifically because you saw the photo on the menu board?				65.3

^1^*BHC* Baltimore Healthy Carry-outs.

### Fidelity

Availability of promoted entrées (e.g. entrée salads, grilled chicken sandwiches) and new healthy sides (e.g. bananas and baked chips) were measured in the four intervention carry-outs to assess fidelity. Promoted entrees were available 92.5% of the time on average (ranging from 91.0 to 96.1%), suggesting high fidelity. Promoted healthy sides were available on 53.8% of the time on average (ranging from 50.0 to 57.4%), suggesting moderate fidelity. Beverages were not measured because they were stocked directly by the beverage vendors rather than by carry-out owners.

Menu boards were appropriately placed with 100% fidelity throughout all phases in the four intervention carry-outs. However, we found that one carry-out did not place posters immediately after the initiation of Phase 1. Interventionists helped owners place the posters the following week and posters remained visible throughout the remainder of the intervention. Overall, posters were visibly posted 97.5% of the time, suggesting high fidelity (Table 3). Lastly, interventionist visits to the carry-outs were completed with 100% fidelity.

## Discussion

Baltimore Healthy Carry-outs (BHC) was one of the first interventions to target the carry-out food environment in low-income urban communities. The study presented here provides evidence that environmental interventions targeting prepared food sources can be successfully implemented, and therefore may be effective in promoting healthy eating in low-income urban areas. Prior food intervention studies have focused primarily on full service restaurants, fast food restaurants, and cafeteria environments and have left carry-out settings unexplored [12,31,32]. By including carry-outs owned by store owners of different ethnicities (African-Americans and Korean-Americans) we sought to find intervention strategies with greater generalizability. Intensive formative research helped the researchers build positive relationships with carry-out owners and implement the intervention successfully [17].

The BHC trial was one of the first prepared food source interventions to perform a thorough process evaluation assessing reach, dose, and fidelity. Since BHC was a community-based study, the proportion of people exposed, known as reach, was difficult to assess because there is no standardized method to measure reach in community settings where individuals cannot be accurately tracked. Total sales were collected to assess the impact of BHC on sales and serve as a proxy indicator for reach. This was a feasible method to determine reach, under the assumption that increased sales would be associated with a higher number of individuals reached. The BHC trial was well implemented with moderate to high dose delivered and high fidelity. Our process evaluation findings were consistent with studies conducted in other settings aimed at modifying the retail food environment [29,30,33]. BHC had a mean promoted entrée availability of 92%, which was higher than 70% seen in the First Nations retail food stores in Northwest Ontario, Canada [33] and 78% seen in Apache reservations in East-central Arizona [30]. We found nearly 100% visibility of the menu boards and posters, while similar studies showed lower poster visibility average ranging from 61.4% to 82% in Apache and Baltimore, respectively [29,30].

During implementation of the BHC trial, a number of lessons were learned (Table 4). One of the most important elements was developing rapport with carry-out owners. We strategically developed intervention strategies that required little to no involvement of the owners during Phase 1. We did not ask owners to add healthier items to their menus in the first phase; instead we used their existing menu list when creating the new board. Placing little obligation on carry-out owners was crucial to initiate a successful intervention in these settings. The importance of establishing strong rapport has been shown in other interventions in prepared food source settings [17,31,34]. Maintaining communication with owners and incorporating their ideas for intervention strategy development enhanced commitment and proved to be key to the success of the intervention. This has also been found in other prepared food source interventions that have targeted educational materials towards restaurant owners in an effort to encourage voluntary participation [23,24,35]. For example, if the owner’s principle language was not English, using greetings in his/her native language or providing materials in the native language helped establish effective communication [36]. Respect for the carry-out owners’ time and independence was also important for developing rapport. For example, BHC researchers waited until all present customers had been served whenever communicating with the owners.

**Table 4:** Lessons learned from the Baltimore Healthy Carry-outs intervention

**Goal**	**Method**	**Outcome**
**Recruitment**	■ Stratifying stores by ethnicity of owners followed by random sampling	■ Recruitment of comparison and intervention carry-outs that could be more accurately compared
	■ Visiting several times before active recruiting	■ Built a rapport and trust with owners
	■ Explaining the purpose of the study	■ Appealed to interest of business owners to contribute to the community
**Develop rapport**	■ Use the same interventionists to visit the carry outs	■ Owners are more likely to trust the individuals they are more familiar with
	■ If applicable, learn and use greetings in Korean	■ Cultural sensitivity is shown to carry out owners
	■ Discuss store benefits for participation (e.g. gift cards, new menu boards) and benefits to researcher team (improved community health)	■ Owners are much more likely to participate in the study if they are given incentives and understand the research purpose
**Motivate owners**	■ Provide owners with supplies required of the intervention	■ Simplified receipt collection process; reduce error in data collection
	■ Provide initial stock of new items	■ Reduce risk of carrying new item
	■ Provide appropriate equipment for food item preparation	■ Owners will not be burdened by additional costs to introduce healthier items and can use the same ingredients, but healthier cooking methods.
**Signage feasibility**	■ Show owners pictures of food items before displaying them on new materials	■ Owners will be more accepting of picture taking when shown how appealing food photos look
	■ Show owners and customers different options for the logo to get buy-in and determine preference	■ Owners and customers more responsive to menu logos
**Low burden strategies**	■ Menu-labeling and posters advertising healthy items	■ Placed little obligation on already busy business owners
	■ Providing high-quality, attractive materials and helping put them up	■ Business owners liked the new materials, which led to higher acceptability

The study had several limitations. First, we may have underestimated availability of healthier promoted entrees and side dishes in the evaluation. This was because items that were not available at the time of the process evaluation were marked as “not stocked”. It is possible that these items may have only been sold out temporarily, and thus the process evaluation may have underestimated the number of healthy items available. Future prepared food source trials may benefit from having more frequent process evaluations and multiple measures of availability before the trial begins. Second, we were not able to visually assess the food storage areas at the carry-outs to verify the stock status of promoted foods. To minimize the burden on carry-out owners, we instead relied on recall. Third, BHC had limited process evaluation data on consumers, particularly concerning reach, when compared to a retail store intervention that focused on customer interaction [29]. Lastly, only 66% of carry-outs approached agreed to participate in BHC, which may have led to sampling bias. We did not inquire as to the reason for not participating. However, it appeared that the owners feared having researchers on their premises would interfere with business practices. Since BHC was a feasibility study, during recruitment we were unable to provide evidence to support that BHC would result in positive sales. Therefore, when recruiting intervention carry-outs in the future, providing positive impact outcomes data may be beneficial.

Overall, the results show effective implementation and suggest that BHC is a culturally acceptable intervention in small, urban prepared food sources. Additional analyses on outcomes (published elsewhere) showed an increase in purchases of healthy items and total revenue among intervention carry-outs [25]. These findings were promptly communicated to the Baltimore City Food Policy Initiative, an inter-governmental collaboration focused on increasing access to healthy foods in food deserts. In recognizing the prevalence of prepared food sources in a concentrated food market setting, the Baltimore City Food Policy Initiative is applying BHC’s intervention strategies to the city’s public markets, with the goal to cover all six of Baltimore’s public markets by 2015 [37].

## Conclusions

The Baltimore Healthy Carry-outs intervention was successfully implemented and provides a model for further research in prepared food sources in low-income, ethnic-minority communities. We recommend that similar future interventions incorporate formative research in the intervention strategy development and conduct a thorough process evaluation to determine how effectively the intervention is employed. Based on the process evaluation outcomes from the BHC intervention, we also recommend building rapport with carry-out owners, making gradual changes to the food environment that place minimal burden on the staff, and incorporating owners’ feedback on the intervention. In addition, working closely with local food policy initiatives throughout the intervention may help develop programs and policies that will have a prominent, lasting positive impact on the food environment.

### Availability of supporting data

The intervention materials supporting the results of this article are available in the Baltimore Healthy Carry-outs repository, http://healthystores.org/projects/baltimore-healthy-carryouts/.

## Abbreviations

BHC: Baltimore healthy carry-outs.

## Competing interests

The authors declare that they have no competing interests. This work was supported by the Center for a Livable Future and the Baltimore Diabetes Research and Training Center.

## Authors’ contributions

SHL and JG conceptualized the study and developed hypotheses. SHL, SG, VH, JJ, MH carried out intervention implementation and data acquisition. SHL and RY analyzed the data. SHL and BB drafted the article. SG, VH, RY, BB, JJ, MH contributed to the interpretation of study findings. All authors read and approved the final manuscript.

## Authors’ information

SG, VH, BB and JJ were with Johns Hopkins Bloomberg School of Public Health at the time of research. SG is currently a Clinical Dietitian at Massachusetts General Hospital Weight Center, Boston, MA; VH is currently a Program Coordinator in Research and Education at the Bladder Cancer Advocacy Network, Bethesda, MD; BB is currently a Ph.D. student and Assistant Policy Analyst at Pardee RAND Corporation, San Jose, CA; RY is currently the Healthy Food Coordinator for Baltimore City Food Policy Initiative, Baltimore, MD; JJ is currently a Ph.D. student at University of North Carolina – Chapel Hill Gillings School of Public Health.

## Pre-publication history

The pre-publication history for this paper can be accessed here:

http://www.biomedcentral.com/1471-2458/13/638/prepub
